# Acute pharmacologic management of myocardial infarction in patients undergoing percutaneous coronary intervention: insights from the Italian nationwide EYESHOT-2 prospective registry

**DOI:** 10.1093/ehjopen/oeaf154

**Published:** 2025-11-25

**Authors:** Marco Zuin, Donata Lucci, Paolo Calabrò, Antonino Nicosia, Emanuele Tizzani, Ciro Mauro, Pier Luigi Temporelli, Lucio Gonzini, Aldo Pietro Maggioni, Massimo Grimaldi, Furio Colivicchi, Domenico Gabrielli, Fabrizio Oliva, Leonardo De Luca

**Affiliations:** Department of Translational Medicine, University of Ferrara, Via Savonarola 9, 0532 Ferrara, Italy; Department of Cardio-Thoraco-Vascular Sciences and Public Health, University of Padova, Via VIII Febbraio 2, 35122 Padua, Italy; Department of Cardiology, Madre Teresa Di Calcutta Hospital, AULSS 6, Ospedali Riuniti Padova Sud, Via Albere 30, 35043 Monselice, Italy; Centro Studi ANMCO, Fondazione per il Tuo cuore, Via Alfonso La Marmora 34, 50121 Firenze, Italy; Division of Cardiology, A.O.R.N. ‘Sant'Anna e San Sebastiano’, Via Ferdinando Palasciano, 81100 Caserta, Italy; Dipartimento CardioNeuroVascolare, ASP Ragusa, Via Dante Alighieri 102, 97100 Ragusa, Italy; Dipartimento di Cardiologia, Ospedale degli Infermi, Via Rivalta 29, 10098 Rivoli, TO, Italy; Division of Cardiology, ‘A. Cardarelli’ Hospital, Via Antonio Cardarelli 9, 80131 Naples, Italy; Division of Cardiac Rehabilitation, Istituti Clinici Scientifici Maugeri, IRCCS, via per Revislate 13, 28013 Gattico-Veruno, Italy; Centro Studi ANMCO, Fondazione per il Tuo cuore, Via Alfonso La Marmora 34, 50121 Firenze, Italy; Centro Studi ANMCO, Fondazione per il Tuo cuore, Via Alfonso La Marmora 34, 50121 Firenze, Italy; Department of Cardiology, General Regional Hospital ‘F. Miulli’, Strada Prov. 127 Acquaviva – Santeramo Km. 4, 70021 Acuqviva delle Fonti (Bari), Italy; U.O.C. Cardiologia Clinica e Riabilitativa, Presidio Ospedaliero San Filippo Neri - ASL Roma 1, Via Giovanni Martinotti 20, 00135 Roma, Italy; Centro Studi ANMCO, Fondazione per il Tuo cuore, Via Alfonso La Marmora 34, 50121 Firenze, Italy; U.O.C. Cardiologia, Azienda Ospedaliera San Camillo Forlanini, Circonvallazione Gianicolense 87, 00152 Roma, Italy; Cardiologia 1-Emodinamica, Dipartimento Cardiotoracovascolare ‘A. De Gasperis’, ASST Grande Ospedale Metropolitano Niguarda, Piazza dell'Ospedale Maggiore 3, 20162 Milano, Italy; S.C. Cardiologia, Fondazione IRCCS Policlinico San Matteo, V.le Camillo Golgi 19, Pavia 27100, Italy

**Keywords:** Acute coronary syndromes, Acute myocardial infarction, Cardiac care units, Percutaneous coronary intervention

## Abstract

**Aims:**

International guidelines recommend specific antithrombotic strategies for acute myocardial infarction (AMI) patients undergoing percutaneous coronary intervention (PCI). However, real-world practice often diverges. This study aimed to assess antithrombotic therapy use in AMI patients admitted to cardiac care units (CCUs) across Italy.

**Methods and results:**

The EmploYEd Antithrombotic Therapies in Patients with Acute Coronary Syndromes Hospitalized in Italian Cardiac Care Units (EYESHOT-2) registry (NCT06316128) is an Italian nationwide, prospective study evaluating AMI management, focusing primarily on PCI-treated patients. Between 1 and 29 February 2024, 2806 patients were enrolled across 183 Italian CCUs. Percutaneous coronary intervention was performed in 83.5% of cases, while 16.5% received conservative treatment. Patients not undergoing PCI were older, more often female, had more comorbidities, and were more frequently diagnosed with NSTEMI. Pre-treatment with dual antiplatelet therapy before angiography was administered in 58.4% of PCI-treated patients. In the cath lab, most received oral P2Y_12_ inhibitors post-procedure. In-hospital outcomes favoured PCI, with lower rates of mortality, re-infarction, and major bleeding. Independent predictors of in-hospital bleeding among PCI patients included older age [odds ratio (OR) 1.05, 95% confidence interval (CI) 1.03–1.07, *P* < 0.0001], prior bleeding (OR 2.38, 95% CI 1.02–5.59, *P* = 0.046), recent surgery (OR 3.47, 95% CI 1.28–9.42, *P* = 0.01), Killip class III/IV (OR 1.75, 95% CI 1.00–3.05, *P* = 0.049), and femoral access (OR 2.85, 95% CI 1.80–4.49, *P* < 0.0001), while, among patients treated conservatively, included history of anaemia (OR 4.83, 95% CI 2.24–10.41, *P* < 0.0001) and peripheral vascular disease (OR 2.75, 95% 1.24–6.12, *P* = 0.01).

**Conclusion:**

This nationwide registry highlights improvements in AMI care in Italy but reveals persistent discrepancies between guideline-recommended and actual practice, underscoring the need for broader adoption of evidence-based strategies.

**Registration:**

URL: http://www.clinicaltrials.gov. NCT06316128.

## Graphical Abstract

**Graphical Abstract:**
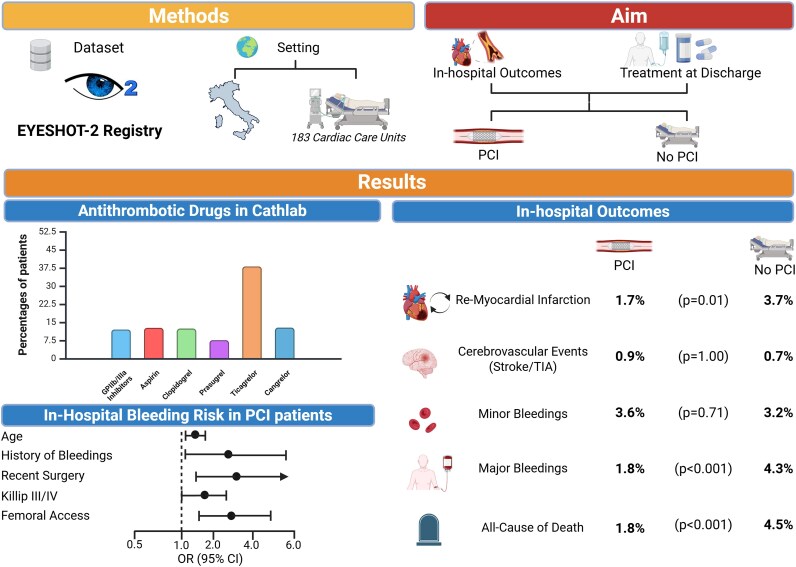

## Introduction

Acute myocardial infarction (AMI) remains a leading cause of morbidity and mortality worldwide, despite significant advances in medical therapies and revascularization techniques.^1–4^ Over the past few decades, management strategies for AMI have evolved, with contemporary guidelines recommending early and aggressive intervention, including percutaneous coronary intervention (PCI), to improve outcomes.^5^ In recent years, several randomized clinical studies have demonstrated that the optimization and correct dosage of antithrombotic therapy can significantly improve the outcomes of patients undergoing PCI during AMI. However, the outcomes observed in these trials may not fully capture the challenges faced in routine clinical practice, where patients often present with a greater burden of comorbid conditions, advanced age, and diverse clinical profiles.^6^ In contrast, observational studies provide valuable insights into the management of AMI in a broader, more heterogeneous population.^7–9^ Observational data are essential in assessing the current treatment landscape, evaluating the implementation of clinical guidelines, and identifying areas of clinical inertia. Furthermore, these studies can uncover unmet clinical needs, such as gaps in therapeutic efficacy, patient adherence, or the necessity for personalized care, which may not be addressed by RCTs alone.

The EmploYEd Antithrombotic Therapies in Patients with Acute Coronary Syndromes Hospitalized in Italian Cardiac Care Units (EYESHOT)-2 registry was created to offer comprehensive, real-world data on the management and clinical outcomes of AMI patients admitted to coronary care units (CCUs) across Italy.^10^ By examining a broad spectrum of patients, ranging from those receiving revascularization strategies to those managed conservatively, the registry aims to identify gaps between clinical practice and recommended guidelines.

The aim of the present analysis was to evaluate the use and optimization of pharmacological treatments during the acute phase and at discharge in AMI patients admitted to Italian CCUs and treated with PCI, a key subgroup in which peri-procedural and discharge strategies can significantly influence outcomes.

## Methods

The EYESHOT-2 registry (ClinicalTrials.gov ID: NCT06316128) was designed as a multicentre, observational, prospective study conducted nationwide across Italy.^10^ The study was endorsed by the National Association of Hospital Cardiologists (ANMCO), which invited all Italian hospitals with CCUs to participate, including university hospitals, general and regional centres, as well as private institutions. The primary aim of EYESHOT-2 was to collect comprehensive, real-world data on the management and clinical outcomes of consecutive patients, with a confirmed diagnosis of AMI [either ST-elevation myocardial infarction (STEMI) or non-ST-elevation myocardial infarction (NSTEMI)] admitted to Italian CCUs over a 1-month period (1–29 February 2024). To balance data completeness with feasibility, patient enrolment was limited to a single month. Representativeness was ensured by including 183 CCUs, evenly distributed across Italian regions and encompassing hospitals of varying complexity levels (hub, spoke, and community centres). Eligible patients were those (i) aged 18 years or older, (ii) admitted to a CCU with a (iii) confirmed diagnosis of AMI, and (iv) presented with ischaemic symptoms lasting at least 10 min, along with either (a) elevated high-sensitivity cardiac troponin (levels above the 99th percentile) and/or (b) electrocardiographic evidence of myocardial ischaemia, including ST-segment elevation in ≥2 contiguous leads (≥0.2 mV in V1−V3 or ≥0.1 mV in other leads), ST-segment depression (≥0.05 mV) in ≥2 contiguous leads, T-wave inversion, or new-onset left bundle branch block.^5^ Conversely, patients were excluded if they had an iatrogenic myocardial infarction (Type 4 or 5 according to the 4th Universal Definition of myocardial infarction),^1^ or if the AMI diagnosis was not confirmed, or if they were participating in a randomized clinical trial. Per the study protocol, all participants were fully informed about the study’s purpose and procedures and were required to provide written informed consent for the anonymous use of their data. The study was approved by the local ethics committees of each recruiting centre and conducted in accordance with the Declaration of Helsinki, as well as national regulatory requirements and laws.

### Data collection and data quality

Data on baseline characteristics, including cardiovascular history, risk factors, and medications at the time of admission, were systematically collected, along with detailed information on the use of cardiac procedures and the type and timing of revascularization (if performed), in-hospital medications, discharge treatments, and major in-hospital clinical events. All data were entered into a standardized electronic case report form (CRF). At each participating site, the principal investigator and co-investigators were responsible for screening all consecutive patients admitted to the CCU. To support compliance, weekly reminders were sent to investigators to reinforce the enrolment of consecutive eligible patients throughout the study period. Data were extracted directly from patient medical records and entered in a secure, web-based electronic CRF, with all data centrally stored at the ANMCO Research Center. The data entry system included an integrated validation process to automatically check for missing data, inconsistencies, and out-of-range values, ensuring the accuracy and reliability of the dataset. A complete list of the enrolling centres is reported in the Supplementary material online, *Appendix*.

### Treatment strategy

For the present analysis, patients were classified into two groups based on the treatment received during their index hospitalization for AMI. The first group (PCI group) included patients who underwent PCI during the index admission for AMI, while the second group (no-PCI) comprised all other patients. The choice of treatment strategy, including vascular access, stent type, use of mechanical circulatory support, and pharmacological therapies, was determined by the treating physicians, in accordance with current clinical guidelines and local protocols.

### Angiographic and procedural assessment

Coronary angiography findings, including the extent of coronary artery disease (CAD), TIMI flow, and lesion characteristics, were recorded. Procedural details included the number and type of stents implanted, treatment of bifurcation lesions, use of rotational atherectomy, and mechanical circulatory support devices. Surgical revascularization data, including the number of arterial grafts and completeness of revascularization, were also collected for patients undergoing coronary artery bypass grafting (CABG).

### Outcome measures

The outcomes of this study included all-cause in-hospital mortality, re-infarction, major and minor bleeding, and cerebrovascular events, stratified by treatment with or without PCI. All events were adjudicated by the local investigators.

### Definition

Myocardial re-infarction during the index hospitalization was defined by the occurrence of new ischaemic symptoms, accompanied by a renewed elevation of myocardial necrosis biomarkers, with or without concurrent electrocardiographic changes. Major bleeding events were classified according to the Bleeding Academic Research Consortium criteria.^11^ Stroke was defined as an acute neurological deficit lasting more than 24 h that impaired the ability to perform daily activities, regardless of imaging confirmation. The occurrence of stroke or transient ischaemic attack (TIA) was categorized as a cerebrovascular event. Hypercholesterolaemia was defined as total cholesterol levels exceeding 240 mg/dL (6.2 mmol/L), LDL cholesterol (LDL-C) levels greater than 160 mg/dL (4.1 mmol/L), or ongoing treatment with lipid-lowering agents. Diabetes mellitus was defined by one or more of the following criteria: glycated haemoglobin ≥ 48 mmol/mol (6.5%), fasting plasma glucose ≥ 7.0 mmol/L, or plasma glucose ≥ 11.1 mmol/L measured during a 2 h oral glucose tolerance test, confirmed on more than one occasion.^12^ All types of diabetes mellitus were included, regardless of the treatment used.

Arterial hypertension was defined as a resting systolic blood pressure greater than 140 mmHg or diastolic blood pressure greater than 90 mmHg, confirmed on at least two separate measurements, or current use of antihypertensive medications.^13^ Renal dysfunction was defined as an estimated glomerular filtration rate below 60 mL/min. Severe chronic obstructive pulmonary disease was defined in accordance with the Global Initiative for Chronic Obstructive Lung Disease criteria, characterized by significant airflow limitation with a post-bronchodilator forced expiratory volume in one second to forced vital capacity ratio of less than 50%, confirmed by previous medical records.^14^

### Statistical analysis

Continuous variables were expressed as mean ± standard deviation (SD) or median with interquartile range (IQR) and compared using the Student’s *t*-test or Mann–Whitney *U* test, as appropriate. Categorical variables were reported as counts and percentages and compared using the *χ*² or Fisher’s exact test. Independent predictors of in-hospital bleeding were evaluated among patients undergoing PCI using a multivariable logistic regression that included variables with a *P* < 0.05 at the univariate analysis. Similarly, a logistic regression analysis was performed among patients managed conservatively (PCI not performed), with a backward selection procedure, given the relatively high number of covariates with respect to the number of bleeding events. Results are presented as odds ratios (ORs) with corresponding 95% confidence intervals (CIs). A two-sided *P* < 0.05 was considered statistically significant. Statistical analyses were performed using SAS statistical software (version 9.4; SAS Institute Inc., Cary, NC, USA). Data completeness was ≥96% for all analysed variables. A complete list of variables with missing data not captured in the registry is reported in Supplementary material online, *Table S1*.

## Results

### Population and clinical settings

From 1 February to 29 February 2024, a total of 2806 Italian patients with confirmed AMI were enrolled across 183 participating CCUs. Among these centres, 76.5% were equipped with interventional facilities, and 69.4% offered 24/7 catheterization laboratory services. Of the enrolled patients, 2343 (83.5%) underwent PCI during the index admission, while the remaining 463 patients (16.5%) did not receive PCI. Among these, 390 (84.2%) patients underwent coronary angiography (87 with an indication for CABG) while 73 (15.8%) patients did not undergo coronary angiography during the index admission.

### Baseline characteristics

Patients who did not undergo PCI were significantly older (72 ± 13 vs. 68 ± 12 years, *P* < 0.0001) and more frequently female (39.1% vs. 24.0%, *P* < 0.0001) compared to those treated with PCI. Non-ST-elevation myocardial infarction was the predominant diagnosis in the no-PCI group (84.7% vs. 46.3%, *P* < 0.0001). Patients in the no-PCI group exhibited a higher prevalence of cardiovascular risk factors and comorbidities, including dyslipidaemia, diabetes mellitus, hypertension, and renal dysfunction. They also had a greater burden of prior cardiovascular disease, with higher rates of previous myocardial infarction, surgical revascularization, heart failure, peripheral artery disease, and prior major bleeding events. In addition, patients not treated with PCI were more frequently on antithrombotic therapy before admission, particularly aspirin, P2Y_12_ inhibitors, dual antiplatelet therapy (DAPT), and oral anticoagulants (*Table 1*).

**Table 1:** Baseline clinical characteristics

	PCI	No PCI	*P* value
	*n* = 2343	*n* = 463	
Age, years, mean ± SD	68 ± 12	72 ± 13	<0.0001
Age ≥75 years, *n* (%)	825 (35.2)	226 (48.8)	<0.0001
Female, *n* (%)	562 (24.0)	181 (39.1)	<0.0001
BMI, kg/m^2^, mean ± SD	26.9 ± 4.3	26.7 ± 4.9	0.30
Diagnosis at admission, *n* (%)			
STEMI	1258 (53.7)	71 (15.3)	<0.0001
NSTEMI	1085 (46.3)	392 (84.7)	
Risk factors and comorbidities, *n* (%)			
Familiar history of CAD (data available for 2546 pts)	508/2154 (23.6)	80/392 (20.4)	0.17
Active smokers	787 (33.6)	100 (21.6)	<0.0001
Dyslipidaemia (data available for 2722 pts)	1187/2271 (52.3)	274/451 (60.8)	0.001
Diabetes mellitus	611 (26.1)	143 (30.9)	0.03
Hypertension	1536 (65.6)	344 (74.3)	0.0003
Renal dysfunction/dialysis	270 (11.5)	102 (22.0)	<0.0001
Severe COPD	206 (8.8)	59 (12.7)	0.008
Active cancer	53 (2.3)	15 (3.2)	0.21
Cardiovascular history, *n* (%)			
Previous stroke/TIA	117 (5.0)	34 (7.3)	0.04
Peripheral artery disease	187 (8.0)	65 (14.0)	<0.0001
History of stable angina	156 (6.7)	37 (8.0)	0.30
History of major bleedings and/or transfusions	51 (2.2)	18 (3.9)	0.03
History of heart failure	97 (4.1)	57 (12.3)	<0.0001
Previous MI	427 (15.2)	136 (29.4)	<0.0001
Previous PCI	467 (19.9)	112 (24.2)	0.04
Previous CABG	77 (3.3)	39 (8.4)	<0.0001
Antithrombotic therapy before admission, *n* (%)			
ASA	649 (27.7)	184 (39.7)	<0.0001
P2Y_12_ inhibitor	185 (7.9)	64 (13.8)	<0.0001
DAPT	102 (4.4)	51 (11.0)	<0.0001
LMWH	22 (0.9)	3 (0.7)	0.54
OAT	157 (6.7)	48 (10.4)	0.006

ASA, acetylsalicylic acid; BMI, body mass index; CABG, coronary artery by-pass grafting; CAD, coronary artery disease; COPD, chronic obstructive pulmonary disease; DAPT, dual antiplatelet therapy; LMWH, low molecular weight therapy; MI, myocardial infarction; NSTEMI, Non-ST-elevation myocardial infarction; OAT, oral anticoagulant therapy; PCI, percutaneous coronary intervention; STEMI, ST-elevation myocardial infarction; TIA, transient ischaemic attack.

Compared to PCI-treated patients, those who did not undergo PCI more often presented with advanced Killip class III–IV (14.2% vs. 6.9%, *P* < 0.0001) and atrial fibrillation/flutter on the first ECG (10.8% vs. 5.6%, *P* < 0.0001). In contrast, cardiac arrest at presentation was more common among patients who underwent PCI (3.6% vs. 1.1%, *P* = 0.0001). Systolic blood pressure, heart rate, and ejection fraction were similar between these two groups. Laboratory findings showed that conservatively managed patients had significantly lower haemoglobin levels (13.1 ± 2.2 vs. 13.7 ± 2.0 g/dL, *P* < 0.0001), higher serum creatinine (1.2 ± 1.2 vs. 1.1 ± 0.8 mg/dL, *P* = 0.008), and lower total cholesterol and LDL-C levels (all *P* < 0.0001). Platelet counts (even though not statistically significant) and triglyceride levels were also modestly lower in the no-PCI group (*Table 2*).

**Table 2:** Haemodynamic parameters and laboratory variables at baseline

	PCI	No PCI	*P* value
	*n* = 2343	*n* = 463	
Killip class III-IV, *n* (%) (data available for 2683 pts)	153/2231 (6.9)	64/452 (14.2)	<0.0001
Cardiac arrest, *n* (%)	84 (3.6)	5 (1.1)	0.0001
SBP, mmHg, mean ± SD	136 ± 25	138 ± 24	0.18
HR, b.p.m., mean ± SD	78 ± 17	80 ± 18	0.43
Ejection fraction, %,mean ± SD	47.7 ± 9.8	48.0 ± 11.5	0.58
AF/flutter at first ECG, *n* (%) (first ECG available for 2727 pts)	128/2282 (5.6)	48/445 (10.8)	<0.0001
Hb, gr/dl, mean ± SD	13.7 ± 2.0	13.1 ± 2.2	<0.0001
Creatinine, mg/dL, mean ± SD	1.1 ± 0.8	1.2 ± 1.2	0.008
Glycaemia, mg/dL, mean ± SD (data available for 2742 pts)	139.0 ± 61.9	135.8 ± 57.3	0.17
Platelet count, ×1000, mean ± SD	240.4 ± 75.6	232.5 ± 83.6	0.06
Total cholesterol, mg/dL, mean ± SD (data available for 2640 pts)	172.5 ± 48.7	161.7 ± 48.1	<0.0001
LDL-C, mg/dL, mean ± SD (data available for 2604 pts)	107.9 ± 43.9	96.1 ± 41.7	<0.0001
Triglycerides, mg/dL median [IQR] (data available for 2631 pts)	104 [77–140]	96 [73–127]	0.007

AF, atrial fibrillation; Hb, haemoglobin; HR, heart rate; LDL, low-density lipoprotein; NSTEMI, Non-ST-elevation myocardial infarction; SBP, systolic blood pressure; STEMI, ST-elevation myocardial infarction.

### Dual antiplatelet therapy before coronary angiography

Among patients treated with PCI, a pre-treatment with DAPT was administered to 1368 patients (58.4%), including 759 (60.3%) of those with a STEMI diagnosis at admission and 609 (56.1%) of those with NSTEMI (*P* = 0.04), respectively (Supplementary material online, *Table S2*).

### Coronary angiography findings

Coronary angiography was performed in 2733 patients, with a predominant use of the radial approach (92.6% overall), which was higher in NSTEMI than STEMI patients (93.6% vs. 91.5%, *P* = 0.03). Regarding CAD severity, single-vessel disease was the most common finding (38.9% overall), observed in 45.3% of STEMI patients and 32.9% of those with NSTEMI (*P* < 0.0001). Multi-vessel disease (involving two or three vessels) was present in 56.3% of cases, with a higher prevalence in NSTEMI compared to STEMI patients (59.2% vs. 53.3%, *P* = 0.002). Angiographically normal coronary arteries were infrequent, found in only 4.8% of patients overall, with a higher occurrence in the NSTEMI group (7.9% vs. 1.4%, *P* < 0.0001). Detailed data on coronary angiography findings and PCI rates, stratified by type of myocardial infarction, are provided in Supplementary material online, *Tables S3* and *S4*.

### In-cath lab antithrombotic management

Among the 2343 patients who underwent PCI, antithrombotic treatment administered in the catheterization laboratory varied substantially. Glycoprotein (GP) IIb/IIIa inhibitors were used in 282 patients (12.0%). Aspirin and clopidogrel were administered in 297 (12.7%) and 290 (12.4%) patients, respectively. Prasugrel was given to 179 patients (7.6%), while ticagrelor was the most used P2Y₁₂ inhibitor, administered in 893 patients (38.1%). Cangrelor was used in 300 patients (12.8%). In the cath lab, most of the patients received i.v. antiplatelet agents during the PCI while oral agents were given after PCI (see Supplementary material online, *Figure S1*).

Regarding parenteral anticoagulation administered in the cath lab, unfractionated heparin was the predominant agent, administered in 2125 patients (90.7%). Low molecular weight heparin (LMWH) and fondaparinux were used infrequently, in 37 (1.6%) and 9 (0.4%) patients, respectively. Data on anticoagulant treatment administered in the cath lab are presented in Supplementary material online, *Tables S5* and *S6* for patients undergoing coronary angiography and for those who also underwent PCI, respectively. The use of intravenous antiplatelet agents administered in the cath lab (GPIIbIIIa inhibitors and cangrelor) (overall, in patients treated and in patients not treated with dual antiplatelet before coronary angiography) among patients who underwent PCI is presented in Supplementary material online, *Table S7*.

### Procedural characteristics in percutaneous coronary intervention–treated patients

**Figure 1:**
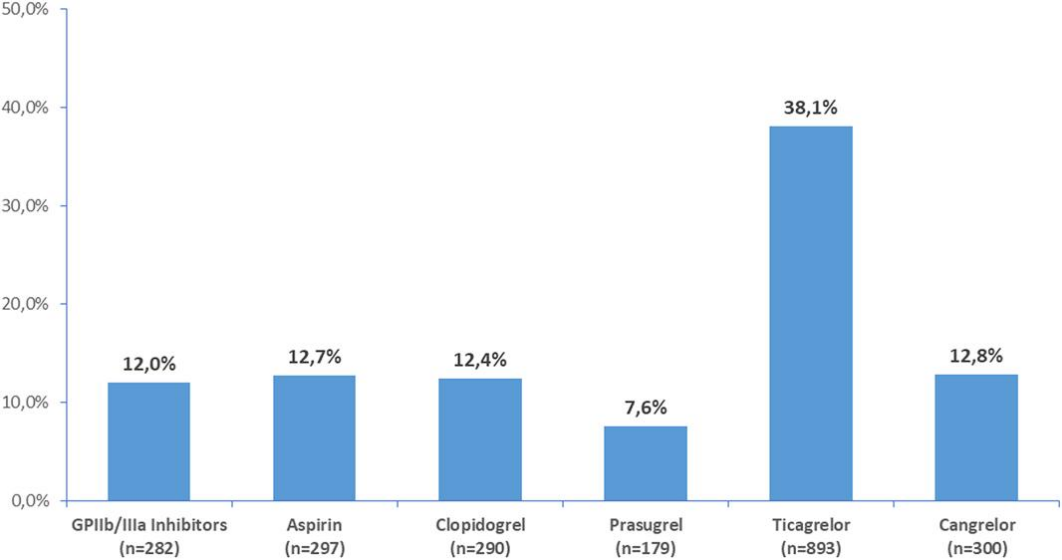
Antithrombotic drugs administered in the cath lab.

Among the 2343 patients who underwent PCI, the majority (91.9%) were treated with drug-eluting stents (DES), with a significantly higher use in patients with STEMI compared to those with NSTEMI (93.9% vs. 89.7%, *P* < 0.001). The mean number of stents implanted was slightly lower in NSTEMI patients compared to STEMI patients (1.7 ± 1.0 vs. 1.8 ± 1.0, *P* = 0.007). Bifurcation lesions were more frequently treated in NSTEMI patients than in those with STEMI (17.1% vs. 11.7%, *P* < 0.001). Baseline TIMI 0/1 flow, indicative of occluded or severely impaired coronary perfusion, was significantly more common in STEMI patients compared to those with NSTEMI (61.2% vs. 27.4%, *P* < 0.0001). The use of rotational atherectomy was generally infrequent but was required more often in NSTEMI patients than in those with STEMI (1.9% vs. 0.5%, *P* = 0.004). During hospital stay, mechanical circulatory support devices, such as intra-aortic balloon pump, microaxial flow pumps, extracorporeal membrane oxygenation, and invasive ventilation, were used significantly more frequently in patients treated with PCI compared to those managed conservatively. Similarly, morphine administration was more common in PCI-treated patients than in those who did not undergo revascularization (9.5% vs. 3.7%; *P* < 0.0001). Complete revascularization was achieved in 75.9% of patients overall, with rates of 74.9% in NSTEMI patients and 76.6% in STEMI patients (*P* = 0.40) (see Supplementary material online, *Table S4*). The percentage use of antithrombotic drugs in-cath lab is showed in *Figure 1*.

### In-hospital outcomes

**Figure 2:**
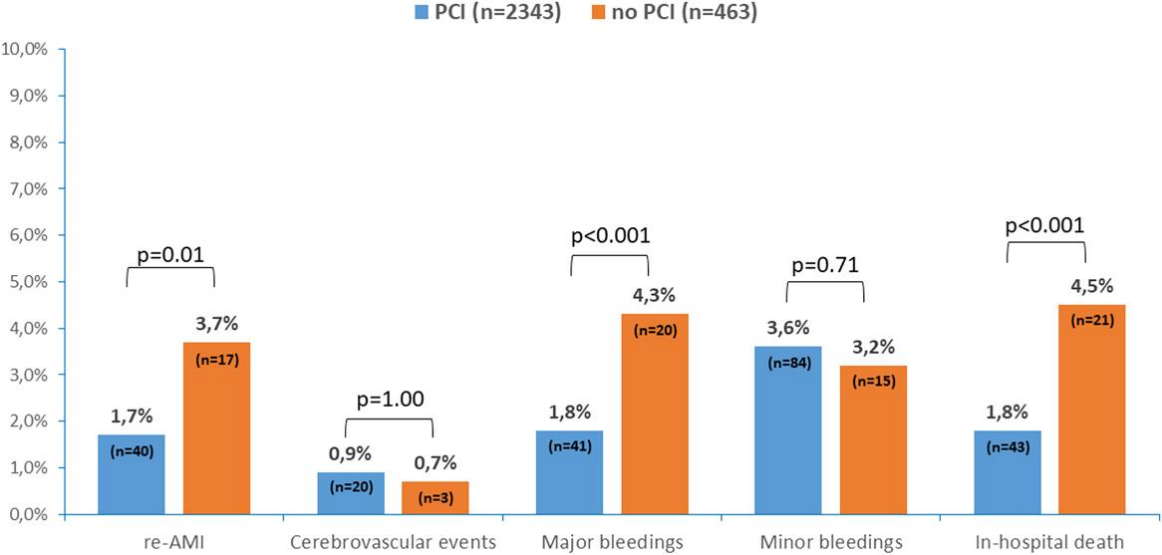
In-hospital outcomes, stratified by patients managed with or without percutaneous coronary intervention. AMI, acute myocardial infarction; PCI, percutaneous coronary intervention.

Median hospital stay was 6 days (IQR 5–9): 6 (IQR 4–9) for NSTEMI and 6 days (IQR 5–9) for STEMI patients (*P* = 0.001). Patients treated with PCI had significantly lower rates of mortality (1.8% vs. 4.5%, *P* = 0.0004), re-infarction (1.7% vs. 3.7%, *P* = 0.01), and major bleeding events (1.8% vs. 4.3%, *P* = 0.0005) compared to those managed without PCI. In contrast, the prevalence of cerebrovascular events and minor bleeding was similar between the two groups (*Figure 2*).

Among patients treated with PCI the administration of GPIIb/IIIa inhibitors, cangrelor, and their combined use was associated with overall bleeding rates of 6.2% (*n* = 17/270), 5.5% (*n* = 16/288), and 16.6% (*n* = 2/12), respectively; furthermore, at multivariable analysis, independent predictors of in-hospital bleeding events included increasing age (OR 1.05, 95% CI 1.03–1.07, *P* < 0.001), history of bleeding (OR 2.38, 95% CI 1.02–5.59, *P* = 0.046), recent surgery (OR 3.47, 95% CI 1.28–9.42, *P* = 0.01), Killip class III/IV (OR 1.75, 95% CI 1.00–3.05, *P* = 0.049), and femoral access for PCI (OR 2.84, 95% CI 1.80–4.48, *P* < 0.001) (*Table 3*).

**Table 3:** Multivariable analysis for the independent predictors of in-hospital bleeding events among percutaneous coronary intervention patients

	Univariate			Multivariable		
	OR	95% CI	*P*	OR	95% CI	*P*
Age	1.06	1.05–1.08	<0.0001	1.05	1.03–1.07	<0.0001
History of bleedings	4.66	2.28–9.54	<0.0001	2.38	1.02–5.59	0.046
Recent surgery	5.32	2.11–13.44	0.0004	3.47	1.28–9.42	0.01
Killip III/IV	3.22	1.95–5.32	<0.0001	1.75	1.001–3.05	0.049
Femoral access for PCI	3.83	2.50–5.86	<0.0001	2.85	1.80–4.49	<0.0001

CI, confidence interval; OR, odds ratio; PCI, percutaneous coronary intervention.

When the multivariable analysis was performed among patients treated conservatively (PCI not executed), only history of anaemia (OR 4.83, 95% CI 2.24–10.41, *P* < 0.0001) and peripheral vascular disease (OR 2.75, 95% 1.24–6.12, *P* = 0.01) resulted independent predictors of in-hospital bleeding events.

### Medical therapy at discharge

**Figure 3:**
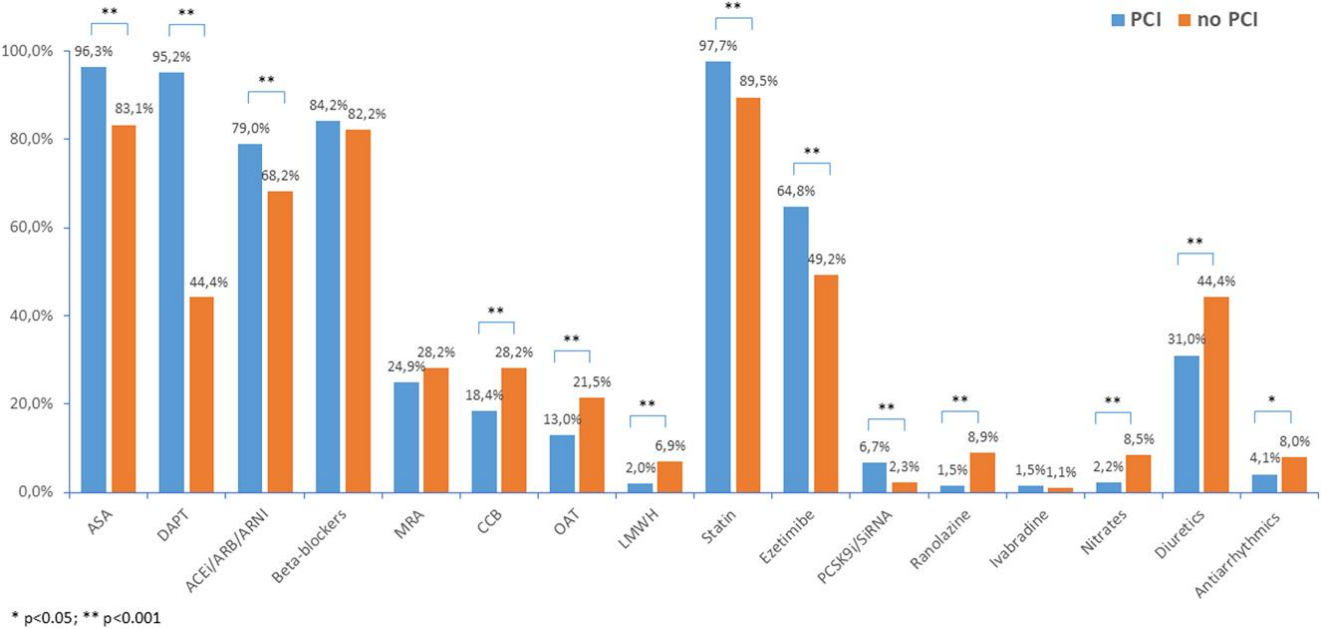
Pharmacological treatment at discharge, stratified according to the type of management (percutaneous coronary intervention vs. conservative management). PCI, percutaneous coronary intervention.

At discharge, most the 2731 patients discharged alive, regardless of treatment strategy, were prescribed guideline-directed therapies. Patients who underwent PCI (*n* = 2294) had a higher prescription rate of DAPT (95.2% vs. 44.4%) and statins (97.7% vs. 89.5%) compared to those managed without PCI (*n* = 437). Similarly, the use of aspirin (96.3% vs. 83.1%) and ezetimibe (64.8% vs. 49.2%) was more frequent in PCI-treated patients. Conversely, a no-PCI strategy was associated with a higher use of oral anticoagulants (21.5% vs. 13%), calcium channel blockers (28.2% vs. 18.4%), diuretics (44.4% vs. 31%), nitrates (8.5% vs. 2.2%), and ranolazine (8.9% vs. 1.5%). The prescription rates of ACE inhibitors/ARBs/ARNI and beta-blockers were generally high in both groups but slightly lower in the no-PCI cohort. The use of PCSK9 inhibitors (PCSK9i), mineralocorticoid receptor antagonists (MRA), ivabradine, low molecular weight heparin, and antiarrhythmics was relatively low overall, though PCSK9 inhibitors showed a higher prescription rate in PCI patients compared to those treated conservatively (5.9% vs. 2.3%, *P* = 0.002) (*Figure 3*).

## Discussion

This nationwide, prospective registry offers an updated and comprehensive overview of the real-world management of AMI in Italian CCUs. Specifically, data from the EYESHOT-2 registry reveal a high utilization of PCI, along with notable differences in clinical characteristics, treatment patterns, and outcomes between patients treated with PCI and those who were not.

Among the 2806 enrolled patients, 83.5% underwent PCI, in line with updated guideline-recommended practices. In contrast, patients who did not receive PCI were characterized by some peculiar characteristics including such as older age, female sex and a greater comorbidity burden, which probably created concerns regarding the peri-procedural risk as well as the bleeding risk associated with DAPT.^15^ These findings mirror those of previous registries and highlight the challenge of extending interventional strategies to patients with frailty, renal dysfunction, and prior cardiovascular events, where the risk–benefit ratio may be less favourable or technically complex.^16–18^ Notably, most no-PCI patients underwent coronary angiography, demonstrating a high level of diagnostic effort even in those not selected for revascularization. The decision to forgo PCI was often due to anatomical complexity, procedural risk, or an indication for surgery, underscoring the need for individualized therapeutic approaches, particularly for high-risk or elderly populations.^19–21^

Regarding the pharmacological management, this analysis shows that the antithrombotic management of AMI patients in Italy generally aligns with current guidelines, though variability exists in both the pre- and in-cath lab phases. In this context, pre-treatment with DAPT—which is not consistently recommended by current guidelines^5^—is used in approximately half of AMI patients undergoing PCI in Italy. This may be due to multiple reasons, such as the perception of benefit of DAPT despite current evidence especially when the times to PCI are estimated as prolonged.^22,23^ Among patients treated with PCI, the use of GPIIb/IIIa inhibitors, cangrelor, and newer P2Y₁₂ inhibitors, such as ticagrelor, was prevalent but not universal.^24–28^ Although the use of intravenous antiplatelet agents was associated with higher bleeding rates,^24^ the multivariable analysis only confirmed established clinical predictors of in-hospital bleeding, including femoral access, prior bleeding history, and Killip class III/IV.^21^ These findings reinforce the importance of individualizing antithrombotic strategies based on patient risk profiles and procedural complexity. The widespread adoption of radial access is notable, exceeding many contemporary European benchmarks and confirming its integration into daily practice.^29^ Drug-eluting stents were used in nearly all PCI cases, with significantly higher rates in STEMI patients. Moreover, despite a higher prevalence of multi-vessel disease among NSTEMI patients, complete revascularization was achieved more frequently in this group, possibly reflecting more elective or staged PCI approaches compared to the often urgent, culprit-lesion-focused interventions in STEMI.^30^

As expected, PCI-treated patients had significantly better in-hospital outcomes, including lower rates of all-cause mortality, re-infarction, and major bleeding events. These findings align with previous randomized trial data and large registries that support early invasive management of AMI.^31–34^ However, residual event rates in the no-PCI group were substantially higher, raising important questions about unmet needs in this subset of patients. While a conservative strategy may be appropriate for selected high-risk individuals, the increased morbidity and mortality observed in this group warrant further exploration of barriers to revascularization, including frailty, logistical delays, or physician-level decision-making biases.^35^ Notably, these findings should not be interpreted as causally attributable to the absence of PCI itself, but rather as indicative of an underlying high-risk phenotype.

At discharge, prescription rates for guideline-directed medical therapy (GDMT) were generally high across the cohort, particularly among PCI-treated patients. However, the underuse of statins, DAPT, and renin–angiotensin–aldosterone system inhibitors in the no-PCI group remains concerning, especially given their proven prognostic benefit independent of revascularization.^35–37^ This under-prescription may stem from concerns about bleeding risk, drug–drug interactions, or therapeutic nihilism in the elderly or patients with multiple comorbidities. Nonetheless, efforts should be made to optimize GDMT even in conservatively managed patients, potentially through a multidisciplinary approach and improved transitional care strategies. Despite robust evidence demonstrating mortality and morbidity benefits of GDMT, even in the absence of revascularization, its implementation remains suboptimal. This gap likely reflects therapeutic inertia, concerns about bleeding risk, and the complexity of managing comorbidities in this population. Strengthening adherence to guideline recommendations represents a key opportunity to improve outcomes in patients managed without revascularization.

Present results highlight the need for continued efforts to bridge the gap between guideline recommendations and clinical practice, especially in the most vulnerable patient populations. Tailored risk stratification tools shared decision-making models, and the expanded use of heart team discussions could help optimize care for patients who are not immediately eligible for PCI. Furthermore, the data underscore the need for consistent use of evidence-based pharmacological therapies at discharge, regardless of the revascularization strategy employed. Future studies are needed to evaluate long-term outcomes and develop interventions aimed at improving adherence to both invasive and medical treatment strategies in real-world AMI patients.

### Strengths and limitations

The primary strength of the EYESHOT-2 registry lies in its real-world design, large sample size, and national representation across various hospital types, which enhances the generalizability of its findings. The prospective design, high data completeness, and rigorous adjudication of outcomes further reinforce the robustness of the results. However, several limitations must be acknowledged. First, the observational nature of the study precludes causal inference. Data on long-term outcomes were not collected, limiting the ability to assess the durability of treatment effects. Additionally, reasons for withholding PCI were not systematically recorded, and despite adjustments, the risk of residual confounding in comparing PCI and no-PCI groups remains. Variability in inter-operator decision-making and procedural techniques may also affect generalizability. Furthermore, our study’s data were limited to the hospitalization period, and while participating centres were instructed to enrol all consecutive AMI patients admitted to the CCU, we could not independently verify the enrolment process due to the lack of administrative auditing, which raises the potential for under-reporting, particularly of early fatal events. Nevertheless, based on data from previous nationwide registries, the enrolment rate is reliable, and selective enrolment at specific sites is unlikely to have significantly impacted the findings. The characteristics of the enrolling centres were consistent with those of Italian CCUs overall. Additionally, all clinical events were adjudicated by local investigators, which may introduce variability in event classification and potential bias. Finally, since the registry exclusively included Italian patients, the generalizability of its findings to a broader population is limited. Despite these limitations, the EYESHOT-2 registry provides valuable insights into the management of AMI patients hospitalized in CCUs across Italy.

## Conclusions

The EYHSHOT-2 registry offers important insights into the current management of AMI patients admitted to the CCU. While substantial progress has been made in in-hospital AMI care over the years, discrepancies between established guidelines and real-world practice persist. In this context, these data present a valuable opportunity to bridge the gap between scientific evidence and guideline recommendations, promoting their more effective integration into clinical practice.

## Lead author biography


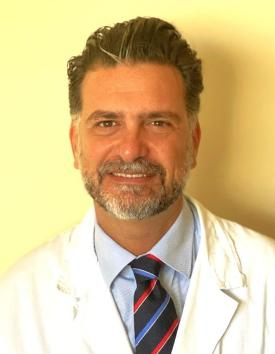


Leonardo De Luca, MD, PhD, FESC, FACC, is the Director of Cardiology at the IRCCS University Hospital San Matteo in Pavia, Italy. He received his MD and PhD from the University La Sapienza of Rome and completed his research training at the University Hospital in Netherlands, Portugal, and USA. He also obtained a master’s degree in intensive cardiac care from the University La Cattolica and in healthcare management from the Luiss Business School of Rome. He is the Vice-President of the Heart Care Foundation—ANMCO, the Governor elect of the American College of Cardiology Italian chapter, and a member of the Research Section of the ESC-ACC (Association of Acute Cardiovascular Care).

## Supplementary Material

oeaf154_Supplementary_Data

## Data Availability

The data that support the findings of this study are available on request from the corresponding author.
