# Supplementary material for: Acute pharmacologic management of myocardial infarction in patients undergoing percutaneous coronary intervention: insights from the Italian nationwide EYESHOT-2 prospective registry
Source: Eur Heart J Open. 2025 Nov 25;5(6):oeaf154. doi: 10.1093/ehjopen/oeaf154 (PMC12686988; doi:10.1093/ehjopen/oeaf154)

**Acute Pharmacologic Management of Myocardial Infarction in Patients Undergoing PCI: Insights from the Italian nationwide EYESHOT-2 Prospective Registry**

Marco Zuin ^1,2,3^, Donata Lucci^4^, Paolo Calabrò^5^, Antonino Nicosia^6^, Emanuele Tizzani^7^, Ciro Mauro^8^, Pier Luigi Temporelli^9^, Lucio Gonzini^4^, Aldo Pietro Maggioni^4^, Massimo Grimaldi^10^, Furio Colivicchi^11^, Domenico Gabrielli^4,12^, Fabrizio Oliva^13^, Leonardo De Luca^14^ on behalf of EYESHOT-2 Investigators**

^1^Department of Translational Medicine, University of Ferrara, Ferrara, Italy; ^2^Department of Cardio-Thoraco-Vascular Sciences and Public Health, University of Padova, Padua, Italy; ^3^Department of Cardiology, Madre Teresa Di Calcutta Hospital, AULSS 6, Ospedali Riuniti Padova Sud, Monselice, Italy; ^4^Centro Studi ANMCO, Fondazione per il Tuo cuore, Firenze, Italy; ^5^Division of Cardiology, A.O.R.N. 'Sant'Anna e San Sebastiano', Caserta, Italy; ^6^Dipartimento CardioNeuroVascolare - ASP Ragusa, Italy; ^7^Dipartimento di Cardiologia, Ospedale degli Infermi, Rivoli (TO), Italy; ^8^Division of Cardiology, "A. Cardarelli" Hospital, Naples, Italy; ^9^Division of Cardiac Rehabilitation, Istituti Clinici Scientifici Maugeri, IRCCS, Gattico-Veruno, Italy; ^10^Department of Cardiology, General Regional Hospital "F. Miulli", Bari, Italy; ^11^U.O.C. Cardiologia Clinica e Riabilitativa, Presidio Ospedaliero San Filippo Neri - ASL Roma 1, Roma, Italy; ^12^U.O.C. Cardiologia, Azienda Ospedaliera San Camillo Forlanini, Roma, Italy; ^13^Cardiologia 1-Emodinamica, Dipartimento Cardiotoracovascolare "A. De Gasperis", ASST Grande Ospedale Metropolitano Niguarda, Milano; ^14^S.C. Cardiologia, Fondazione IRCCS Policlinico San Matteo, Pavia, Italy

***Supplementary Files***

[**Appendix** 2](#_Toc209113323)

[**Supplementary Table 1.** Variables with missing data not captured in the registry. 6](#_Toc209113324)

[**Supplementary Table 2.**  Use of individual P2Y₁₂ inhibitors before coronary angiography in patients treated with dual antiplatelet therapy (DAPT) before coronary angiography, among patients who underwent percutaneous coronary intervention (PCI). 7](#_Toc209113325)

[**Supplementary Table 3.** Vascular access used for the coronary angiography and numbers of disease coronary arteries, stratified by type of myocardial infarction. 8](#_Toc209113326)

[**Supplementary Table 4.** Procedural characteristics. 10](#_Toc209113328)

[**Supplementary Table 5.** Anticoagulant pretreatment (prior to cath-lab access) among patients who underwent coronary angiography. 11](#_Toc209113329)

[**Supplementary Table 6.** Anticoagulant pretreatment (prior to cath-lab access) among patients who underwent percutaneous coronary intervention. 12](#_Toc209113330)

[**Supplementary Table 7.** Use of intravenous antiplatelet agents, (overall, in patients treated with dual antiplatelet before coronary angiography, and in those not treated with dual antiplatelet before coronary angiography), among patients who underwent percutaneous coronary intervention.. 13](#_Toc209113331)

[**Supplementary Figure 1.** Relative percentage of antiplatelet agents administered in the CathLab in relation to PCI. 14](#_Toc209113332)

# **Appendix**

*Steering Committee*

Leonardo De Luca (Chairman), Furio Colivicchi (Co-Chairman), Domenico Gabrielli (Co-Chairman), Fabrizio Oliva (Co-Chairman), Claudio Cavallini, Michele Massimo Gulizia, Sergio Leonardi, Aldo Pietro Maggioni, Alice Sacco, Fortunato Scotto Di Uccio, Serafina Valente.

*Coordinating Center*

Heart Care Foundation, ANMCO Research Center, Florence, Italy (Donata Lucci, Francesca Bianchini, Lucio Gonzini, Andrea Lorimer, Laura Sarti, Aldo Pietro Maggioni).

*Participating Centers and Investigators*

Catanzaro, P.O. "Pugliese" - A.O.U. "Renato Dulbecco", UTIC-Emodinamica e Cardiologia Interventistica (V Pascale, G Galiano Leone, VA Ciconte, A Ferraro); Erice, P.O. S. Antonio Abate di Trapani, UOC Cardiologia, UTIC ed Emodinamica (G Geraci, D Zabbia, L Priolo); Avellino, A.O.R.N. San Giuseppe Moscati, UO Cardiologia/UTIC 'D. Rotiroti' (F Lanni, E Di Lorenzo, F Nicastro); Caserta, A.O. S. Anna e S. Sebastiano, UO Cardiologia d'Emergenza con UTIC (P Calabrò, F Gragnano, E Golia); Ragusa, Ospedale Giovanni Paolo II, UOC Cardiologia-UTIC (A Nicosia, A Sanfilippo, SM Bono); Rivoli, Ospedale Degli Infermi, SC Cardiologia (E Tizzani, D Celentani, G Zanda); Napoli, A.O.R.N. Cardarelli, UO Cardiologia con UTIC (C Mauro, G Carpinella, F La Rocca); Viterbo, Ospedale Belcolle, UOSD UTIC Polo (F Serra, P Achilli, K Sensi); Desio, Ospedale Pio XI, UOC Cardiologia (F Achilli, D Saltafossi, C Bersano); Parma, A.O.U. Parma, UOC Cardiologia (G Paoli, R Bonura, R Giacalone); Rimini, Ospedale Infermi, UO Cardiologia (F Ottani, V Moriconi, F Contorni); Arezzo, Ospedale San Donato, UOC Cardiologia (L Bolognese, A Sabini, A Sori); Bologna, Ospedale Policlinico S. Orsola-Malpighi, UO Cardiologia-Galiè (A Corsini, M Garofalo, F Barberini); Cagliari, ARNAS G. Brotzu, Cardiologia con UTIC (M Corda, G Binaghi, L Semeraro) Palermo, ARNAS P.O. Civico e Benfratelli, UOC UTIC (IM Smecca, F Dispensa, N Giunta); Pescara, Ospedale Civile Dello Spirito Santo, Cardiologia con UTIC (M Di Marco, A D'Alleva, D Di Clemente); Piacenza, Ospedale Civile 'Guglielmo Da Saliceto', UOC Cardiologia e UTIC (D Aschieri, S Ferraro, V Pelizzoni); Roma, Ospedale Sandro Pertini, UOC Cardiologia (A Granatelli, P Trambaiolo, I Figliuzzi); Torino, Ospedale Maria Vittoria, Cardiologia (MRT-MV) (B Demichelis); Treviso, Ospedale Ca' Foncello, UOC Cardiologia (C Cernetti, N Gasparetto, L Franceschini); Aversa, P.O. Moscati, UOC Cardiologia-UTIC (L Fattore, V Santillo, A Chianese); Boscotrecase, Ospedale Civile di Boscotrecase, UTIC Cardiologia (M Macrì, A Varricchio, C Del Giudice); Foggia, Policlinico Riuniti, SC Cardiologia Universitaria-UTIC (ND Brunetti, E Guerriero, VA Paciello); Monserrato, A.O.U. Cagliari Policlinico Monserrato, SC Cardiologia UTIC Emodinamica (R Montisci, MF Marchetti); Prato, Ospedale Santo Stefano, UO Cardiologia (A Toso, E Di Vincenzo, E Vignini); Bari, Ospedale San Paolo, Cardiologia-UTIC (P Caldarola, N Locuratolo, D Rutigliano); Bolzano, Ospedale Centrale Bolzano, Cardiologia e Prove Funzionali (R Cemin, A Mahfouz); Genova, Ospedale Villa Scassi - ASL 3 Ligure, SC Cardiologia UTIC (L Pastorino, F Mainardi, F Beccaria); Magenta, Ospedale Civile Fornaroli, UO Cardiologia (A Russo, L Striuli, T De Ferrari); Palermo, AOR Villa Sofia-Cervello P.O. Villa Sofia, UOC Cardiologia e UTIC e Emodinamica-Villa Sofia (MS Scaccianoce, V Zingone, M Lo Cascio); Roma, Ospedale Santo Spirito, UOC Cardiologia (A Cuono, A Michielon, E Sollazzo); Eboli, Ospedale Maria SS. Addolorata, UO Cardiologia UTIC (A Catalano, M Scalcione, L Santimone); Lecce, Ospedale Vito Fazzi, UOC Cardiologia-UTIC ed Emodinamica (S Marazia, G Chiarello, G Colonna); Livorno, Ospedali Riuniti, UOC Cardiologia e UTIC (R Gistri, G Terlizzese, G Mansi); Pavia, Fondazione IRCCS Policlinico San Matteo, UOC Cardiologia 1 (M Portolan, R Camporotondo, V Spirlì); Reggio Emilia, P.O. Santa Maria Nuova - AUSL RE IRCCS, SOC Cardiologia Ospedaliera (F Manca, F Piroli, M Azzarone); Ancona, Ospedali Riuniti, SOD Cardiologia Ospedaliera e UTIC (GP Perna, E Paolini, L Piangerelli); Firenze, AOU Careggi, Interventistica Cardiologica Strutturale (C Di Mario, F Caniato, G Conciarelli); Sassari, Ospedale SS. Annunziata, Cardiologia Clinica ed Interventistica (G Casu, F Bilotta, P Franca); Savona, P.O. Levante - Ospedale San Paolo, SC Cardiologia Levante (M Buscemi, A Somaschini, P Bellone); Ferrara, Arcispedale Sant'Anna, UO Cardiologia (G Guardigli, P Cimaglia, FM Verardi); Pietra Ligure, P.O. Ponente - Ospedale Santa Corona, SC Cardiologia Ponente (S Moshiri, A Nicolino, MA Corona); Pistoia, Ospedale San Jacopo, SOC Cardiologia (E Pedemonte, T Mariani, F Ciatti); Ravenna, Ospedale Civile Santa Maria Delle Croci, UOC Cardiologia (A Rubboli, S Vecchio, D Girolami); Vicenza, Ospedale Civile San Bortolo, UOC Cardiologia (G Barbati, L Mogentale); Bologna, Ospedale Maggiore, UOC Cardiologia (G Casella, S Zagnoni, F Sciarra); Legnano, ASST Ovest Milanese - P.O. di Legnano, Cardiologia e Unità Coronarica (F Poletti, F Mori); Monza, Fondazione IRCCS San Gerardo Dei Tintori - Ospedale San Gerardo, UOC Cardiologia (F Del Furia, A Mauro, DC Corsi); Potenza, AOR San Carlo - Ospedale San Carlo, SSD Terapia Intensiva Cardiologica (P Innelli, L Spera, GA Paternò); Sanremo, Presidio Ospedaliero, UO Cardiologia (F Ferrari, N Pingelli); Trento, Ospedale Santa Chiara, Divisione di Cardiologia (P Zeni); Vimercate, Nuovo Ospedale di Vimercate, SC Cardiologia e UTIC (M Stucchi, ML Lazzarotti); Lavagna, Ospedale del Tigullio Polo Lavagna, SC Cardiologia e UTIC (G Parodi, V Oliva); Milano, Osp. San Luca - Istituto Auxologico Italiano IRCCS, Area Critica Cardiologica (S Blengino, D Mariani); Palermo, AOR Villa Sofia-Cervello P.O. Cervello, UO Cardiologia-Cervello (V Polizzi, MG Carmina); Palermo, Ospedale G.F. Ingrassia, Cardiologia-UTIC (P Levantino, P Cottone); Torino, Ospedale Mauriziano Umberto I, SC Cardiologia (G Musumeci, S Civera); Treviglio, Ospedali Treviglio-Caravaggio, Cardiologia (A Cardile, D Rubino); Brescia, ASST Spedali Civili, Cardiologia (E Gorga, G Guida); Empoli, Ospedale San Giuseppe, Cardiologia (B Bellandi, M Solari); Maddaloni, Casa Di Cura San Michele, UO Cardiologia (M Pepe, S Severino); Nocera Inferiore, P.O. Umberto I, UOC Cardiologia-UTIC (M Pacileo, A D'Andrea); Orbassano, AOU San Luigi Gonzaga, SCDO Cardiologia (M Bianco, A Chinaglia); Palermo, AOU Policlinico P. Giaccone, UOC Cardiologia (D Puccio, F D'Angelo); Salerno, AOU S. Giovanni Di Dio-Ruggi D'Aragona, SSD UTIC (A Ravera, A Campanile); San Donà Di Piave, Ospedale Civile, UOC Cardiologia (C Canali); Crema, Ospedale Maggiore, UO Cardiologia e UTIC (M Marino); Foligno, Nuovo Ospedale San Giovanni Battista, SC Cardiologia (C Andreoli, E Biscottini); Nuoro, Ospedale San Francesco, Cardiologia-UTIC (MRS Pisano, S Bonano); Udine, POU Santa Maria della Misericordia, SOC Cardiologia (M De Biasio, M Imazio); Chieti, Ospedale Policlinico SS. Annunziata, UOC Cardiologia-UTIC (M Zimarino, F Radico); Ivrea, Ospedale Civile, SC Cardiologia (E Lonni, F Troiano); La Spezia, Ospedale S. Andrea, SC Cardiologia (A Menozzi, I Puccetti); Pesaro, P.O. San Salvatore, Cardiologia e UTIC (M Cottini); Torino, AOU Città della Salute e della Scienza, SC Cardiologia U (S Frea, PP Bocchino); Avola, Ospedale G. Di Maria, Cardiologia UTIC (M Mizzi, L Sirugo); Campobasso, Ospedale A. Cardarelli, UO Cardiologia e UTIC (AR Colavita); Mirano, Presidio Ospedaliero Di Mirano, UOC Cardiologia (S Saccà, E Artuso); Seriate, Ospedale Bolognini, Cardiologia e Unità Coronarica (C Angeletti, A Costalunga); Siena, AOU Senese Ospedale S. Maria Alle Scotte, Cardiologia (S Valente, N Ghionzoli); Acquaviva Delle Fonti, Ospedale Miulli, UOC Cardiologia-UTIC (M Grimaldi, F Troisi); Bagno A Ripoli, Ospedale Santa Maria Annunziata, SOS Cardiologia Clinica (A Ibrahim, MF Crociani); Cirié, POR Cirié-Lanzo Presidio Cirié, SC Cardiologia (V Infantino, G Senatore); Cuneo, A.O. Santa Croce e Carle, SC Cardiologia (R Rossini, F Maiellaro); Milano, IRCCS Centro Cardiologico Monzino, Terapia Intensiva Cardiologica (UTIC) (M Moltrasio); Olbia, Ospedale Giovanni Paolo II, UTIC-Cardiologia (G Sabino, L Becciu); Piombino, Ospedale Villamarina, Cardiologia e UTIC Piombino Elba (L Frediani, G Borelli); Roma, Ospedale San Camillo, UOC Cardiologia (RL Putini, N Bruno); San Giovanni Rotondo, Fondazione IRCCS Ospedale Casa Sollievo Della Sofferenza, UOC Emodinamica e Cardiologia (C Vigna, G De Luca); Brindisi, Ospedale Perrino, UOC Cardiologia (GP Giorda, MI Gioia); Lodi, Ospedale Maggiore di Lodi, UOC Cardiologia (G Bertazzoli, G Caetani); Milano, ASST Ospedale Metropolitano Niguarda, Cardiologia 1-Emodinamica (A Sacco, G Viola); Roma, Aurelia Hospital, UOC Cardiologia (F Tomai, M Michisanti); San Bonifacio, Ospedale G. Fracastoro, UOC Cardiologia (M Anselmi, A Cima); Venezia-Mestre, Ospedale Dell'Angelo, UOC Cardiologia (A Cutolo, A Bellin); Verduno, Ospedale Pietro e Michele Ferrero, Cardiologia e UTIC (V Bovolo, M Pegoretti); Domodossola, Ospedale San Biagio, SOC Cardiologia VCO (A Lupi); Forlì, Ospedale G.B. Morgagni-L. Pierantoni, UO Cardiologia (R Carletti, A Marinelli); Messina, IRCCS Centro Neurolesi Bonino Pulejo - P.O. Piemonte, Cardiologia e UTIC (S Quattrocchi, G Falanga); Milano, Ospedale L. Sacco, SC Cardiologia (M Viecca, A Autieri); Novara, AOU Maggiore della Carità, SSVD UTIC (L Rossi, L Airoldi); Perugia, A.O. Perugia, SC Cardiologia (M Del Pinto, A Broccatelli); Pescia, Ospedale SS. Cosma e Damiano, SOS Cardiologia (S Stroppa, GCS Magnaghi); Pozzuoli, Ospedale Santa Maria Delle Grazie, UO Cardiologia - UTIC con Emodinamica (L Cavuto); Roma, Ospedale Madre Giuseppina Vannini, UOC Cardiologia (D Manzo, E Belmonte); San Donato Milanese, IRCCS Policlinico San Donato, UO Cardiologia con UTIC (F Bedogni, A Vella); Vallo Della Lucania, Ospedale San Luca, UO UTIC-Cardiologia (A Aloia, N Ragosa); Verona, AOUI di Verona, UO Cardiologia (D Prati, M Magistri); Altamura, Ospedale Della Murgia - Fabio Perinei, SC Cardiologia-UTIC (P Scicchitano, F Massari); Ariccia, Ospedale Dei Castelli, UOC Cardiologia e UTIC (M D'Agostino, M Tancredi); Bari-Carbonara, Ospedale Di Venere, UOC Cardiologia (MV Bonfantino, V De Luca); Napoli, A.O.R.N. Ospedale Dei Colli - P.O. Monaldi, UOC Cardiologia-UTIC (G Petrillo); Ponderano, Ospedale Degli Infermi, SC Cardiologia (A Rognoni, E Battistini); Rieti, P.O. San Camillo De Lellis, UOC Cardiologia (A Kol, M Schiavoni); Roma, Policlinico Casilino, UOC Cardiologia (ME Donahue, G Zimbardo); Taranto, Casa Di Cura Villa Verde, Cardiologia (AN John); Arzignano, Ospedale Civile, UOC Cardiologia (C Bilato, V Battisti); Cefalù, Fondazione G. Giglio, UO Cardiologia (D Armata, M Giovino); Massa, Ospedale Apuane, UO Cardiologia-UTIC (G Arena, A Bini); Montebelluna, Ospedale San Valentino, UOC Cardiologia (C Condello, F Brun); Nola, P.O. S. Maria Della Pietà, UO Cardiologia e UTIC (R America, L Nunziata); Tricase, A.O. Cardinale G. Panico, UO Cardiologia-UTIC (M Colopi, L Zezza); Bari, AOU Policlinico, UOC Cardiologia Ospedaliera (C D'Agostino, MI Pansini); Chivasso, Ospedale Civico, SC Cardiologia (S Panella, E Franco); Giugliano In Campania, P.O. San Giugliano, UO Cardiologia-UTIC (G Napolitano, E Arezzi); Milano, ASST Santi Paolo e Carlo-P.O. San Carlo, Cardiologia-UCC (S Lucreziotti, EMP Madonini); Modena, Ospedale Civile di Baggiovara, UO Cardiologia (S Sansoni, E Bastia); Ancona, IRCCS INRCA, UO Cardiologia/UTIC/Telecardiologia (R Antonicelli, D Caraceni); Cesena, Ospedale M. Bufalini, UO Cardiologia-UTIC (CM Iannettone, D Bernucci); Cinisello Balsamo, Ospedale Edoardo Bassini, SC Cardiologia-UCC ASST Nord Milano (Bassini-SSG) (M Pirondini, A Lesce); Fidenza, Ospedale Civile, UOC Cardiologia-UTIC (C Galizia, F De Rosa); Milano, Fondazione IRCCS Ca' Granda, Ospedale Maggiore Policlinico, UOC Malattie Cardiovascolari (S Carugo, L Barbieri); Rho, Ospedale Civile, UO Cardiologia (G De Angelis, E Lisi); Torino, Ospedale San Giovanni Bosco, SC Cardiologia (M Bertaina); Bentivoglio, Ospedale di Bentivoglio, UO Cardiologia Pianura (A De Lorenzis, A Lombardi); Camaiore, Nuovo Ospedale Versilia, SC Cardiologia (J Del Meglio, A Christou); Castellammare Di Stabia, Ospedale San Leonardo, UO Cardiologia-UTIC (F Minicucci, G Mattiello); Castellaneta, Ospedale Di Castellaneta, SC Cardiologia (F Clemente); Cecina, Ospedale Civile Bassa Val Di Cecina, UO Cardiologia e UTIC (E Venturini, MF Fambrini); Gravedona, Ospedale Moriggia Pelascini, UO Cardiologia (A Amato, M Revera); Guastalla, Ospedale Civile Di Guastalla, SOS Cardiologia e Riabilitazione Card Area Nord (A Navazio, E Guerri); Gubbio, Ospedale Gubbio-Gualdo Tadino, UO UTIC e Cardiologia (EA Capponi, D Cosmi); Matera, Ospedale Madonna Delle Grazie, SSD UTIC (MA Clemente, C Morea); Ome, Istituto Clinico San Rocco, UO Cardiologia (AM Lanzone, LM Culot); Reggio Calabria, Grande Ospedale Metropolitano - Osp Riuniti Bianchi Melacrino, UOC Cardiologia e UTIC (F Lucà); Chiari, ASST Franciacorta - Presidio Chiari, Cardiologia/UTIC (C Conti); Città Di Castello, P.O. Città Di Castello, UO Cardiologia/UTIC (L Marinacci, D De Ritis); Mazara Del Vallo, Ospedale A. Ajello, UOC Cardiologia con UTIC (M Meschisi, M Gabriele); Polla, P.O. Luigi Curto, UO Cardiologia-UTIC (S Saponara, R Citro); Roma, Ospedale Isola Tiberina, UOC Cardiologia Diagnostica Interventistica-UTIC (M Ruzzolini, AM Leone); Rozzano, Istituto Clinico Humanitas-IRCCS, Cardiologia Clinica, Interventistica e UCC (LF Bertoldi, M Briani); Sesto San Giovanni, IRCCS Multimedica, UO Cardiologia (A Antonelli, T Staine); Frattamaggiore, Ospedale San Giovanni Di Dio, UO Cardiologia-UTIC (F Piemonte, C Lirato); Montevarchi, Ospedale Valdarno S. Maria Della Gruccia, UO Malattie Cardiovascolari (G Falsini, A Di Florio); Scorrano, Ospedale Ignazio Veris Delli Ponti, UOC Cardiologia-UTIC "E. Vilei" (CA Greco, AL Romano); Cava De' Tirreni, Ospedale S. Maria Incoronata Dell'Olmo, UOC Cardiologia-UTIC (M Malinconico); Gavardo, Ospedale Civile 'La Memoria', UO Cardiologia (I Papa); Napoli, Ospedale Del Mare, UO Cardiologia con UTIC-Emodinamica (B Tuccillo); Palermo, Maria Eleonora Hospital, Cardiologia (V Pernice); Sessa Aurunca, P.O. San Rocco, UOC Cardiologia-UTIC (L Di Lorenzo); Torino, Ospedale Martini, Cardiologia (MRT-MV) (L Coda); Voghera, Ospedale Civile, UO Cardiologia (R Osti); Lentini, P.O. Lentini, UOSD Cardiologia con UTIC (V Crisci); Merate, Ospedale San Leopoldo Mandic, Cardiologia-UCC (F Bruni); Poggibonsi, Ospedale Dell'Alta Val D'Elsa, UOSD Cardiologia-UTIC (M Lucidi); Sassuolo, Ospedale di Sassuolo, Cardiologia (PA Ceresoli); Augusta, Ospedale E. Muscatello, UO Cardiologia-UTIC (G Licciardello); Borgomanero, Ospedale SS. Trinità, SC Cardiologia (PS Dellavesa); Castelnuovo Di Garfagnana, Ospedale Santa Croce, UOSD Cardiologia e di Continuita Assist. (PR Mariani); Cernusco Sul Naviglio, Ospedale di Cernusco Sul Naviglio, UO Cardiologia e UCC (G Macca); Civitanova Marche, P.O. di Civitanova Marche, SC Cardiologia (V Viozzi); Feltre, P.O. Santa Maria Del Prato, UOC Cardiologia-UTIC (C Piergentili); Firenze, Ospedale Santa Maria Nuova, Cardiologia (G Ciriello); Lugo, Ospedale Civile, UO Servizio Cardiologia (G Ricci Lucchi); San Gavino Monreale, Ospedale Nostra Signora Di Bonaria, UOC Cardiologia e UTIC (MC Mulè); Saronno, P.O. di Saronno, UOC Cardiologia (D Nassiacos); Bergamo, Humanitas Gavazzeni, UO Cardiologia (D Cao); Casale Monferrato, Ospedale Santo Spirito, SC Cardiologia (F Nardi); Genova, Ospedale P.A. Micone, SC Cardiologia-UTIC (E Ricciardi); Roma, European Hospital, UOC Cardiologia Interventistica (T Salatino); Santa Maria Capua Vetere, Ospedale San Giuseppe e Melorio, UOC Cardiologia UTIC (P Iodice); Taranto, Ospedale SS. Annunziata, SC Cardiologia-UTIC (V Le Rose); Verbania, Ospedale Castelli, SOC Cardiologia VCO (A Lupi).

# **Supplementary Table 1.** Variables with missing data not captured in the registry.

| **Items** | **%** |
| --- | --- |
| Family history of coronary artery disease | 9.3 |
| Respiratory rate | 15.6 |
| Left ventricular ejection fraction | 4.9 |
| Total cholesterol | 5.9 |
| Low-density lipoprotein cholesterol | 7.2 |
| Triglycerides | 6.2 |
| Dyslipidemia | 3.0 |
| Killip Class | 4.4 |
| Glycemia | 2.3 |

# **Supplementary Table 2.** Use of individual P2Y₁₂ inhibitors before coronary angiography in patients treated with dual antiplatelet therapy (DAPT) before coronary angiography, among patients who underwent percutaneous coronary intervention (PCI). STEMI: ST-elevation myocardial infarction; NSTEMI: Non-ST elevation myocardial infarction.

|  | All  (n=1368) | STEMI  (n=759) | NSTEMI  (n=609) | p |
| --- | --- | --- | --- | --- |
| Clopidogrel, n (%) | 358 (26.2) | 121 (15.9) | 237 (38.9) | <0.0001 |
| Ticlopidine, n (%) | 12 (0.9) | 6 (0.8) | 6 (1.0) | 0.70 |
| Prasugrel, n (%) | 119 (8.7) | 88 (11.6) | 31 (5.1) | <0.0001 |
| Ticagrelor, n (%) | 915 (66.9) | 561 (73.9) | 354 (58.1) | <0.0001 |

**Supplementary Table 3.** Vascular access used for the coronary angiography and numbers of disease coronary arteries, stratified by type of myocardial infarction.

STEMI: ST-elevation myocardial infarction; NSTEMI: Non-ST elevation myocardial infarction.

|  | **Overall**  **N=2733** | **STEMI**  **N=1319** | **NSTEMI**  **N=1414** | **p** |
| --- | --- | --- | --- | --- |
| Radial Approach, n (%) | 2531 (92.6%) | 1207 (91.5) | 1324 (93.6) | 0.03 |
| Femoral Approach, n (%) | 202 (7.4) | 112 (8.5) | 90 (6.4) | 0.03 |
| Coronary arteries disease, n (%)  Normal Arteries  1 vessel  2 vessel  3 vessel | 131 (4.8)  1062 (38.9)  820 (30.0)  720 (26.3) | 19 (1.4)  597 (45.3)  396 (30.0)  307 (23.3) | 112 (7.9)  465 (32.9)  424 (30.0)  413 (29.2) | <0.0001 |

**Supplementary Table 4.** Procedural characteristics. DES: Drug eluting stent; PCI: Percutaneous coronary intervention; STEMI: ST-elevation myocardial infarction; NSTEMI: Non-ST elevation myocardial infarction.

|  | **Overall**  **N=2343** | **STEMI**  **N=1258** | **NSTEMI**  **N=1085** | **p** |
| --- | --- | --- | --- | --- |
| PCI with DES, n (%) | 2154 (91.9) | 1181 (93.9) | 973 (89.7) | <0.001 |
| DES, mean±SD | 1.7±1.0 | 1.7±1.0 | 1.7±1.0 | 0.007 |
| Bifurcation lesions, n (%) | 332 (14.2) | 147 (11.7) | 185 (17.1) | <0.001 |
| Baseline TIMI 0/1, n (%)  *(Available for 2277 pts)* | 1037 (45.5) | 748/1223(61.2) | 289/1054 (27.4) | <0.0001 |
| Rotablator, n (%) | 27 (1.2) | 6 (0.5) | 21 (1.9) | 0.004 |
| Complete Revascularization, n (%) | 1777 (75.9) | 964 (76.6) | 813 (74.9) | 0.40 |
| Peri-procedural complications, n (%) | 106 (4.5) | 57 (4.5) | 49 (4.5) | 1.00 |
| FFR, n (%) | 47 (2.0) | 19 (1.5) | 28 (2.6) | 0.18 |
| IVUS/OCT, n (%) | 168 (7.2) | 61 (4.9) | 107 (9.9) | <0.0001 |
| Throbectomy, n (%) | 183 (7.8) | 159 (12.6) | 24 (2.2) | <0.0001 |

**Supplementary Table 5.** Anticoagulant pretreatment (prior to cath-lab access) among patients who underwent coronary angiography. DAPT: Dual antiplatelet therapy; DOAC: Direct oral anticoagulants; LMWH: Low-molecular-weight heparin; UFH: Unfractionated heparin; OAC: Oral anticoagulant; VKA: Vitamin K antagonist. STEMI: ST-elevation myocardial infarction; NSTEMI: Non-ST elevation myocardial infarction.

|  | **All**  **n=2733** | **STEMI**  **n=1319** | **NSTEMI**  **n=1414** | **p** |
| --- | --- | --- | --- | --- |
| UFH, n (%) | 1244 (45.5) | 843 (63.9) | 401 (28.4) | <0.0001 |
| LMWH, n (%) | 427 (15.6) | 105 (8.0) | 322 (22.8) | <0.0001 |
| Fondaparinux, n (%) | 273 (10.0) | 7 (0.5) | 266 (18.8) | <0.0001 |
| Bivaluridin, n (%) | 8 (0.3) | 3 (0.2) | 5 (0.4) | 0.73 |
| OAC (VKA/DOAC), n (%) | 239 (8.7) | 85 (6.4) | 154 (10.9) | <0.0001 |
| DAPT, n (%) | 1538 (56.3) | 791 (60.0) | 747 (52.8) | 0.0002 |
| GpIIb/IIIa inhibitors, n (%) | 110 (4.0) | 91 (6.9) | 19 (1.3) | <0.0001 |

**Supplementary Table 6.** Anticoagulant pretreatment (prior to cath-lab access) among patients who underwent percutaneous coronary intervention. DAPT: Dual antiplatelet therapy; DOAC: Direct oral anticoagulants; LMWH: Low-molecular-weight heparin; UFH: Unfractionated heparin; OAC: Oral anticoagulant; VKA: Vitamin K antagonist. STEMI: ST-elevation myocardial infarction; NSTEMI: Non-ST elevation myocardial infarction.

|  | **All**  **(n=2343)** | **STEMI**  **(n=1258)** | **NSTEMI**  **(n=1085)** | **p** |
| --- | --- | --- | --- | --- |
| UFH, n (%) | 1146 (48.9) | 804 (63.9) | 342 (31.5) | <0.0001 |
| LMWH, n (%) | 322 (13.7) | 98 (7.8) | 224 (20.7) | <0.0001 |
| Fondaparinux | 211 (9.0) | 6 (0.5) | 205 (18.9) | <0.0001 |
| Bivaluridin, n (%) | 6 (0.3) | 3 (0.2) | 3 (0.3) | 1.0 |
| OAC (VKA/DOAC), n (%) | 187 (8.0) | 78 (6.2) | 109 (10.1) | 0.0006 |
| DAPT, n (%) | 1368 (58.4) | 759 (60.3) | 609 (56.1) | 0.04 |
| GpIIb/IIIa inhibitors, n (%) | 105 (4.5) | 88 (7.0) | 17 (1.6) | <0.0001 |

**Supplementary Table 7.** Use of intravenous antiplatelet agents, (overall, in patients treated with dual antiplatelet before coronary angiography, and in those not treated with dual antiplatelet before coronary angiography), among patients who underwent percutaneous coronary intervention. DAPT: Dual antiplatelet therapy. STEMI: ST-elevation myocardial infarction; NSTEMI: Non-ST elevation myocardial infarction.

|  | **All**  **(n=2343)** | **STEMI**  **(n=1258)** | **NSTEMI**  **(n=1085)** |  |
| --- | --- | --- | --- | --- |
| GpIIb/IIIa inhibitors in cath-lab, n (%) | 282 (12.0) | 235 (18.7) | 47 (4.3) | <0.0001 |
| Abciximab | 22 (7.8) | 18 (7.7) | 4 (8.5) | 0.84 |
| Eptifibatide | 19 (6.7) | 13 (5.5) | 6 (12.8) | 0.10 |
| Tirofiban | 241 (85.5) | 204 (86.8) | 37 (78.7) | 0.15 |
| Bolus, n (%) | 264 (93.6) | 222 (94.5) | 42 (89.4) | 0.23 |
| Intracoronary bolus, n (%) | 67 (23.8) | 54 (23.0) | 13 (27.7) | 0.49 |
| GpIIb/IIIa inhibitors in cath-lab (on 1368 patients pretreated with DAPT, 759 STEMI, 609 NSTEMI), n (%) | 170 (12.4) | 144 (19.0) | 26 (4.3) | <0.0001 |
| GpIIb/IIIa inhibitors in cath-lab (on 975 patients not pretreated with DAPT, 499 STEMI, 476 NSTEMI), n (%) | 112 (11.5) | 91 (18.2) | 21 (4.4) | <0.0001 |
| Mean time infusion of GpIIb/IIIa inhibitors in cath-lab, minutes | 120 [119-150] | 120 [120-158] | 120 [115-138] | 0.17 |
| Cangrelor in cath-lab, n (%) | 300 (12.8) | 199 (15.8) | 101 (9.3) | <0.0001 |
| Cangrelor in cath-lab (on 1368 patients pretreated with DAPT, 759 STEMI, 609 NSTEMI), n (%) | 114 (8.3) | 75 (9.9) | 39 (6.4) | 0.02 |
| Cangrelor in cath-lab (on 975 patients not pretreated with DAPT, 499 STEMI, 476 NSTEMI), n (%) | 186 (19.1) | 124 (24.9) | 62 (13.0) | <0.0001 |

# **Supplementary Figure 1.** Relative percentage of antiplatelet agents administered in the CathLab in relation to PCI. ASA: Aspirin; CVG: Coronary Angiogram; GPI: Inhibitors; PCI: Percutaneous coronary Intervention.


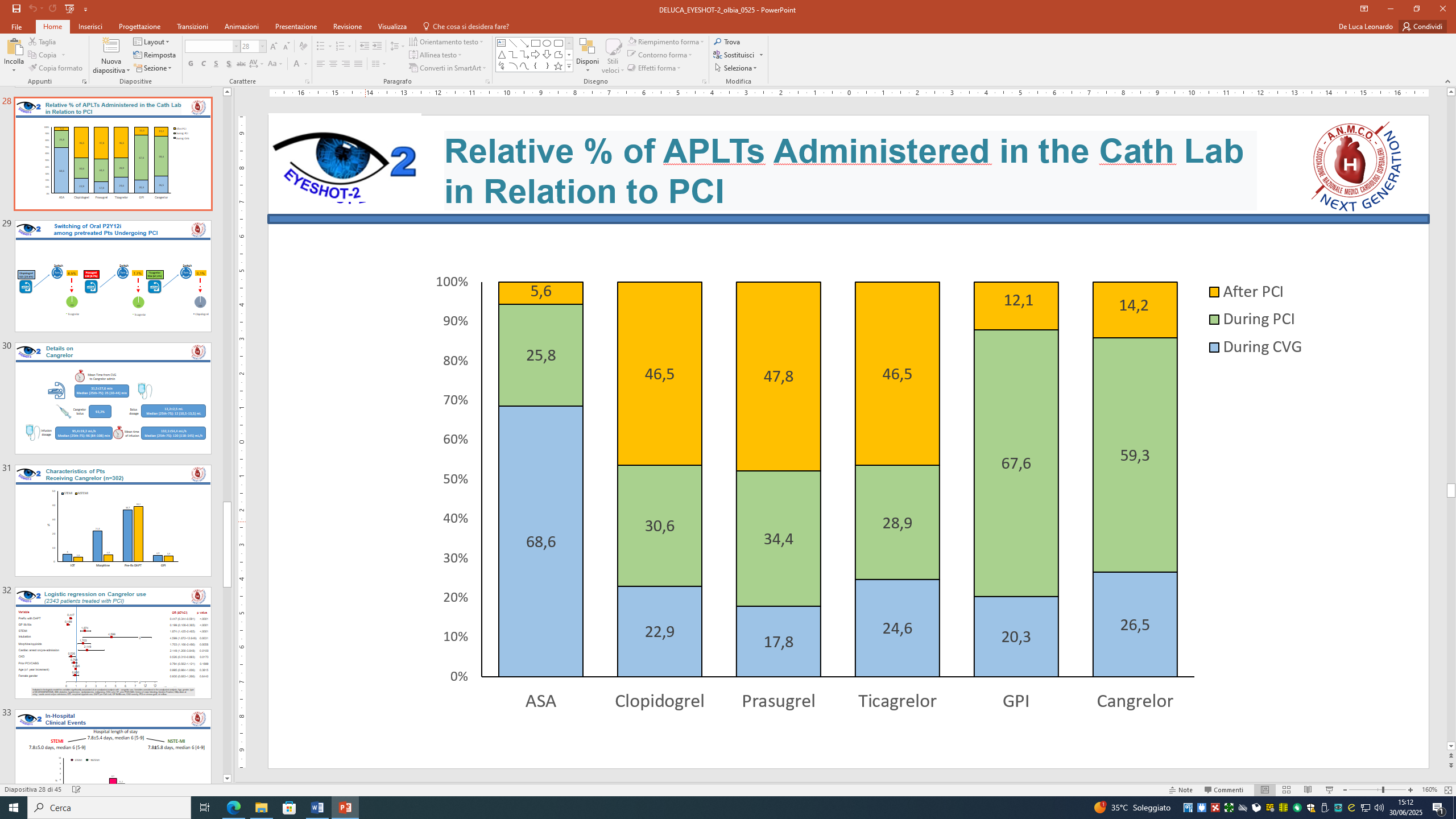

Supplement: oeaf154_Supplementary_Data [file oeaf154_supplementary_data.docx]
